# A systematic review and meta-analysis of the effects of continuous glucose monitoring compared to self-monitoring of blood glucose on glycemic control and maternal and infant outcomes in women with gestational diabetes mellitus

**DOI:** 10.1186/s12884-026-09769-9

**Published:** 2026-08-08

**Authors:** Lin Zheng, Run Tian, Lulu Zhang, Annuo Liu, Peng Wang

**Affiliations:** 1 School of Nursing, Anhui Medical University, 81 Meishan Road, Hefei, Anhui Province China; 2 Department of Maternal and Child Health, West China School of Public Health and West China, Fourth Hospital Sichuan University, Chengdu, Sichuan China

**Keywords:** Gestational diabetes mellitus(GDM), Intervention, Continuous glucose monitoring, Self-monitoring of blood glucose, Glucose control, Pregnant outcome

## Abstract

**Background:**

Continuous glucose monitoring (CGM) compared to self-blood glucose monitoring (SMBG) has been a controversial topic regarding glycemic control in pregnant women with gestational diabetes mellitus (GDM), as well as maternal and infant outcomes. The aim of this study was to assess and compare the effectiveness of CGM and SMBG in managing glycemic control and perinatal outcomes among homebound pregnant women with GDM.

**Methods:**

A comprehensive search was conducted across five databases—PubMed, Web of Science, Medline, Embase, and the Cochrane Library—commencing on July 15, 2024. We included four randomized controlled trials (RCTs) that compared CGM with SMBG in patients with GDM. The primary outcomes assessed were gestational weight gain (GWG), glycated hemoglobin(HbA1c), and the number of people treated with hypoglycemic drugs. Participants were excluded if they had received any glucose-lowering medication prior to enrollment. A meta-analysis of interventions on the basis of at least two studies with 95% confidence intervals and an assessment of heterogeneity via the I² statistic were performed.

**Results:**

The results of the study showed that CGM was significantly associated with a decrease in the number of women with excessive GWG and an increase in the number of GWG-appropriate (RR = 0.57; 95% CI: 0.41, 0.80; I² = 0.0%; *P* = 0.001), (RR = 1.55; 95% CI: 1.19, 2.03; I² = 0.0%; *P* = 0.001). However, GWG-insufficiency was not significantly different (RR = 1.03; 95% CI: 0.47, 2.26; I² = 0.0%; *P* = 0.948). Real-time continuous glucose monitoring (rt-CGM) may offer superior effectiveness in glycemic control management compared with SMBG (SMD=-0.70; 95%CI=-1.07, -0.34; *P* = 0.000; I^2^ = 0.0%), whereas retrospective continuous glucose monitoring (r-CGM) demonstrates greater effectiveness in reducing infant birth weight (SMD=-0.30; 95%CI=-0.54, -0.06; *P* = 0.018; I^2^ = 8.6%). No significant differences were observed in the other perinatal outcomes.

**Conclusion:**

CGM may be more effective than SMBG in preventing excessive weight gain and maintaining appropriate gestational weight in women with GDM. Moreover, rt-CGM appears to offer superior glycemic control compared with SMBG. r-CGM may be more effective in reducing neonatal birth weight.

**Trial registration:**

The PROSPERO registration number is CRD42024571538.

**Supplementary Information:**

The online version contains supplementary material available at https://doi.org/10.1186/s12884-026-09769-9.

## Why was this study conducted?

The effects of continuous glucose monitoring (CGM) on glycemic control and perinatal outcomes in patients with clinically diagnosed GDM remain inconclusive and are still subject to ongoing debate.

## What are the key findings?

CGM is more effective than self-monitoring of blood glucose (SMBG) in preventing excessive gestational weight gain (GWG) and maintaining its stability. Real-time continuous glucose monitoring (rt-CGM) may offer superior effectiveness in glycemic control management compared with SMBG, whereas retrospective continuous glucose monitoring(r-CGM) may demonstrate greater effectiveness in reducing infant birth weight. No significant differences were observed in the other perinatal outcomes.

## What does this study add to what is already known?

This study addresses the controversial issue of the role of CGM in gestational weight management in GDM patients. This is the first study to compare the specific effects of CGM versus SMBG on perinatal outcomes in GDM patients and to further stratify the results on the basis of different types of CGM, despite previous studies that focused on comparisons between CGM and BGM in various populations.

## Introduction

The global incidence of gestational diabetes has significantly increased, rising from 6% in 1977 to 14% in 2021 [1, 2]. Gestational diabetes mellitus (GDM) traditionally refers to abnormal glucose tolerance with onset or first recognition during pregnancy [3]. In late pregnancy, impaired function of pancreatic β-cells coupled with a marked reduction in insulin sensitivity leads to an insufficient ability of the body to meet the elevated insulin demands of pregnancy [4, 5]. This metabolic dysregulation contributes to an increased risk of adverse maternal and neonatal outcomes [6]. The proportion of pregnancies complicated by GDM is positively correlated with an increased risk of developing type 2 diabetes mellitus (T2DM). Additionally, women with a history of GDM have a 63% higher risk of cardiovascular disease (CVD) compared to women without GDM [4]. Offspring of women with GDM are at increased risk of neonatal hypoglycemia and lifelong risk of obesity [4]. These findings further emphasize the importance of both the prevention and management of GDM in women, both before pregnancy and during pregnancy [7].

Currently, the management of glucose control in pregnant women with GDM involves a multifaceted approach, including stringent obstetric glucose monitoring, dietary and physical activity modifications, and the use of insulin and/or oral hypoglycemic agents when necessary [8]. Continuous glucose monitoring (CGM) is an advanced technology for glucose monitoring and management. CGM measures interstitial glucose levels continuously via a small sensor inserted under the skin. This technique captures dynamic glucose profiles and can identify postprandial and nocturnal hyperglycemia that may be missed by traditional glucose monitoring methods [9, 10]. It provides the ability to capture a complete picture of glucose [11]. Furthermore, it is able to help patients prevent adverse glycemic events and guide pharmacological interventions [9, 12]. Moreover, self-monitoring of blood glucose (SMBG), one of the most widely utilized methods for routine blood glucose monitoring in clinical settings, involves obtaining single-point blood glucose measurements through fingertip blood sampling. This technique is favored for its convenience and ease of use [13]. The ongoing clinical debate surrounding the effectiveness of the two interventions in glycemic management across different diabetic populations remains a critical issue.

CGM was originally implemented for glucose control in individuals with type 1 diabetes mellitus (T1DM). A meta-analysis conducted by Teo et al. demonstrated that CGM was significantly more effective in reducing HbA1c levels than SMBG alone [14–16]. As CGM has been increasingly utilized in populations with diabetes due to its effective glucose control, a meta-analysis by Wilkie et al. demonstrated that, compared with SMBG, CGM offers significant advantages in reducing HbA1c levels in both insulin-treated and non-insulin-treated individuals with T2DM [17–20]. However, to date, no study has provided robust evidence demonstrating the superiority of CGM over SMBG alone in terms of glucose control and perinatal outcomes in the GDM population.

An increasing body of evidence has demonstrated the beneficial effects of both CGM and SMBG in improving perinatal outcomes in GDM, including macrosomia, neonatal birth weight, and GWG [21–24]. To the best of our knowledge, no comprehensive meta-analysis has yet been conducted to evaluate the comparative effectiveness of CGM versus SMBG on perinatal outcomes in GDM patients. Among the studies included in the meta-analysis by García-Moreno et al., blood glucose monitoring (BGM) was used as a control group. However, blood glucose monitoring(BGM) is widely used in clinical practice to assess glycemic control status and evaluate the therapeutic efficacy of glucose-lowering interventions. Common methods include SMBG, point-of-care testing (POCT) in hospitals, HbA1c, glycated albumin (GA), and CGM [25], which may yield heterogeneous results [26]. In another report, Chang et al.‘s meta-analysis incorporated a heterogeneous population consisting of patients with GDM, T1DM and T2DM. Although the studies included diverse diabetic populations, no subgroup analyses specifically focused on GDM were performed. This lack of population-specific stratification may introduce confounding variables, potentially undermining the validity of the findings related to GDM-specific outcomes [27]. While the systematic review by Yu et al. provided a descriptive summary of CGM and SMBG in relation to perinatal outcomes in GDM, the studies included in the review were subject to biased sample sizes and study designs and did not undergo quantitative pooling [28]. Moreover, a systematic review conducted by García-Moreno et al. Demonstrated that CGM was significantly associated with GWG outcomes in pregnant women with GDM compared to controls [26]. In contrast, Chang et al. found no statistically significant effect of CGM on GWG outcomes in their study [27].

This systematic review and meta-analysis aim to address the following research questions: (1) To compare the effectiveness of CGM versus SMBG in managing different levels of GWG and (2) to compare the effectiveness of glucose control and perinatal outcomes between various types of CGM and SMBG while also investigating potential confounding factors.

## Materials and methods

Following the protocol registered in PROSPERO (CRD42024571538), we conducted a comprehensive systematic review and meta-analysis. This study adheres to the Preferred Reporting Items for Systematic Reviews and Meta-Analyses (PRISMA) guidelines, ensuring rigorous methodological and reporting standards.

### Information sources and search strategy

**Fig. 1:**
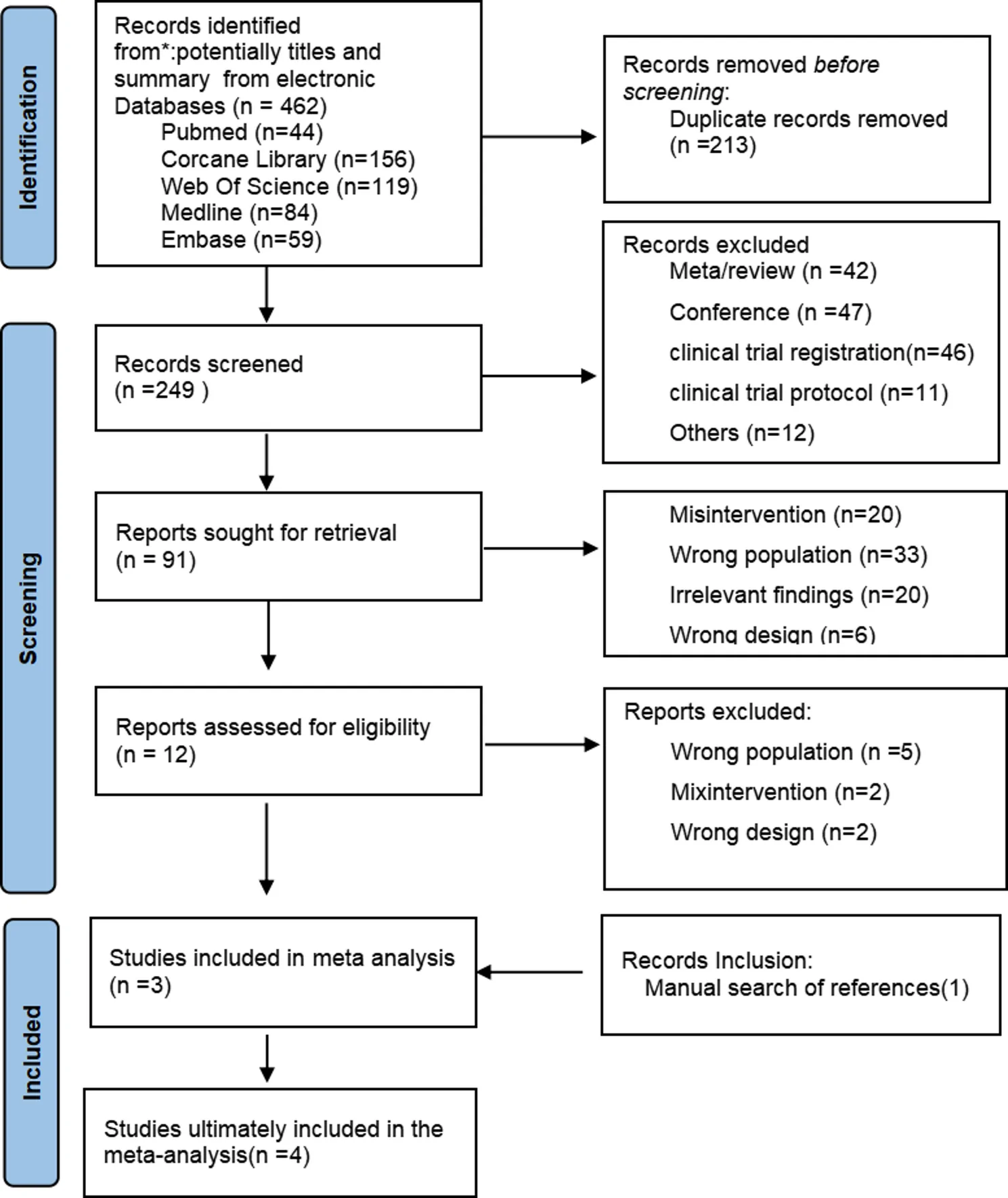
Literature screening flow chart

We integrated Medical Subject Headings (MeSH) with free-text terms to include the following keywords relevant to the study topic: ‘Blood Glucose Self-Monitoring [MeSH]/Continuous Glucose Monitoring’, ‘Diabetes, Gestational [MeSH],’ ‘fetal-outcome* [MeSH]’, and ‘randomized controlled trial’ [Publication Type]. On the basis of these criteria, we systematically searched five databases: PubMed, Web of Science, Embase, the Cochrane Library, and Medline. From 462 potentially relevant articles identified through the initial search, we included 3 articles that met our eligibility criteria. Additionally, we screened the references of these included articles and identified one additional relevant study (by Lin Zheng and Peng Wang), resulting in a total of 4 RCT studies included in our analysis. One study was excluded because of a lack of reporting on the primary outcome variable [29]. The literature screening process was conducted between July 15 and July 20, 2024. See Fig. 1 and Additional file 1 for more details.

### Inclusion and exclusion criteria

The inclusion criteria were as follows: (1) studies meeting the criteria for an RCT; (2) RCT studies involving pregnant women diagnosed with GDM according to different diagnostic criteria; (3) studies comparing continuous glucose monitoring during pregnancy or the postpartum period with self-monitoring of glucose, with a total sample size exceeding 50; (4) studies focusing on perinatal outcomes for pregnant women and neonates. The exclusion criteria were as follows: (1) systematic reviews, meta-analyses, study protocols, trial registries, conference abstracts, letters, and papers lacking relevant information; (2) studies involving individuals with preexisting type 1 or type 2 diabetes mellitus; (3) participants were excluded if they had received any glucose-lowering medication prior to enrollment; (4) studies not reporting the required primary outcome variable; and (5) studies not published in English.

### Study selection

After removing duplicates, two authors (Lin Zheng and Peng Wang) independently screened the titles and abstracts (first step) and reviewed the full texts (second step) applying to preestablished inclusion and exclusion criteria. Data extraction and validation were conducted by one researcher(Lin Zheng), with any discrepancies resolved through consultation with a third party (Annuo Liu). Literature screening and management were conducted via Zotero (Clarivate Analytics).

### Data extraction

The baseline data extracted included maternal age, pregnancy BMI, oral glucose tolerance test (OGTT) (0, 1, 2 h), HbA1c, high-density lipoprotein (HDL), low-density lipoprotein (LDL), and gestational age at birth. The outcomes included the following: (1) primary outcomes: GWG, HbA1c and the number of people treated with hypoglycemic drugs; (2) secondary outcomes: macrosomia, large for gestational age (LGA), small for gestational age (SGA), hyperbilirubinemia, neonatal intensive care unit(NICU) admission, neonatal hypoglycemia, Apgar score at 5 min, and birth weight [30, 31]. While the study primarily focused on pregnancy outcomes, postpartum data, such as macrosomia, neonatal hypoglycemia, and birth weight, were included to explore the broader impact of short-term CGM monitoring on maternal and neonatal health up to the early postpartum period.

### Assessment of the risk of bias of the included studies

Two researchers (Lin Zheng) and (Peng Wang) independently assessed the risk of bias in these studies via the Cochrane risk of bias assessment tool. The assessment included selection bias, reporting bias, performance bias, detection bias, attrition bias, and other potential biases. Each category was classified as ‘low risk’, ‘high risk’, or ‘uncertain risk’. In instances of disagreement regarding the risk of bias, a third researcher (Lulu Zhang) was consulted to resolve discrepancies through consensus. The risk assessment process was completed on July 30, 2024.

### Statistical methods

The data were analyzed via Stata 17.0 software (version 17.0, Cochrane Collaboration Network). A *P* value < 0.05 was considered statistically significant for overall effect differences. Continuous variables were analyzed using means and standard deviations (SDs). The standardized mean difference (SMD) was calculated to compare the differences between the two groups, with SMD defined as the difference in means divided by the pooled standard deviation [32], whereas dichotomous variables are expressed as risk ratios (RRs) and 95% confidence intervals (CIs). Heterogeneity was assessed via the I² statistic. Given the limitations in sample size, we opted to apply a random effects model for all studies, irrespective of the observed heterogeneity levels. This approach was chosen to provide more conservative estimates and mitigate potential biases associated with fixed effects modeling. Restricted maximum likelihood (REML) was chosen as the analysis method. For studies with more than 50% heterogeneity, the source of heterogeneity was investigated through sensitivity analysis or subgroup analysis.

### Definitions and classification of interventions and outcomes

#### CGM (r-CGM and rt-CGM)

Retrospective continuous glucose monitoring (r-CGM) involves the patient wearing a CGM sensor for a defined period, after which glucose data are downloaded and retrospectively analyzed in collaboration with a healthcare professional [33]. In contrast, real-time continuous glucose monitoring (rt-CGM) utilizes a subcutaneous sensor that continuously measures glucose levels in interstitial fluid at 5-minute intervals, providing real-time data and alerts for hyperglycemia and hypoglycemia [34, 35].

#### GWG

According to the National Academy of Medicine (NAM) guidelines, recommended GWG is categorized based on pre-pregnancy body mass index (BMI) as follows: underweight, 12.5–18 kg; normal weight, 11.5–16 kg; overweight, 7–11.5 kg; and obesity, 5–9 kg [36].

## Results

### Study characteristics

The baseline values of each trial were subgrouped, and forest plots demonstrated high consistency and low heterogeneity among the studies (Additional file 2). All four intervention studies were conducted in a home setting. Two studies provided preintervention counseling focused on low-glycemic indices and low-saturated fat diets, with one of these also incorporating recommendations for moderate physical activity [37, 38]. The remaining two studies involved preintervention dietary guidance along with diabetes education [39, 40]. Therefore, it can be inferred that the lifestyle guidance content provided for GDM does not exhibit significant variability across the included RCTs. Three studies were consistent in the timing of SMBG observations (fasting, premeal, and postprandial), whereas one study had separate observations before, after, and at bedtime. The study populations of three trials were classified as nonrisk groups for GDM [37, 39, 40], whereas one trial included a high-risk group for GDM for analysis [38]. The diagnostic criteria for GDM included the IADPSG criteria (25%), the Finnish Diabetes Association criteria (25%) and the ADA criteria (50%) [41–43]. In addition, glucose measurements obtained via the SMBG method were utilized to calibrate the CGM system within the CGM group. Conventional SMBG techniques were employed during periods without CGM monitoring. In the studies included in our analysis, CGM was implemented exclusively for short-term monitoring, while SMBG was consistently applied throughout the entire pregnancy. For further details, refer to Table 1.

**Table 1:** Characteristics of the included randomized controlled trial

Author / year	Country	Gestational weeks	GDM criteria	intervention site	Sample size	Types (CGM)	Exclusion Criteria	Blood glucose target (mmol/L)	CGM group			CGM+SMBG/SMBG group
									Usage Duration	Monitoring Frequency	Brand	Monitoring frequency
Kirsimarja K.,2007	FI	22–34 week	NA	Home	73;EG;36;CG;37; (1:1)	r-CGM	NA	FBG < 5.5	9 days (3 days at weeks 0, 4, and 8)	3 times	Medtronic	4 times/day
								PBG < 7.8				
Qiong,2016	CN	24–28 week	ADA	Home	120;EG;51;CG;55; (1:1)	r-CGM	Pregestational T1DM or T2DM.	FBG < 5.5	Average 48–72 h (on weekdays)	Not specified	Gold Medtronic MiniMed	4 times/day
								PBG(1 h) < 8.6				
								PBG(2 h) < 7.2				
M.Y.,2023	CN	24–28 week	ADA	Home	154;EG;62;CG;62; (1:1)	r-CGM	Diabetes mellitus, previous GDM treatment, infection, or severe comorbidities.	FBG ≤ 5.3	Average 47.4 ± 2.5 h(a week)	Not specified	Medtronic MiniMed CGMS^®^	4–5 times/day
								PBG(1 h) ≤ 7.8				
								PBG(2 h) ≤ 6.7				
Alfadhli,2016	KSA	24–28 week	IADPSG	Home	130;EG;60;CG;62; (1:1)	rt-CGM	Preexisting diabetes, multiple pregnancies, chronic diseases, or drugs affecting pregnancy.	FBG ≤ 5.2	3–7 days	Once	Medtronic MiniMed	4 times/day
								PBG(1 h) ≤ 7.8				
								PBG(2 h) ≤ 6.7				

*FI* Finland, *CN* China, *KSA* Kingdom of Saudi Arabia, *OGTT* Oral Glucose Tolerance Test, *CGMS* Continuous Glucose Monitoring System, *SMBG* Self-Monitoring of Blood Glucose, *IADPSG* International Association of Diabetes and Pregnancy Study Groups, *ADA* American Diabetes Association, *EG* Experimental Group, *CG* Control Group, *PBG* Postprandial glucose, *FBG *fasting blood glucose, *T1DM* Type 1 Diabetes Mellitus, *T2DM* Type 2 Diabetes Mellitus

### Primary outcome

#### GWG

**Fig. 2:**
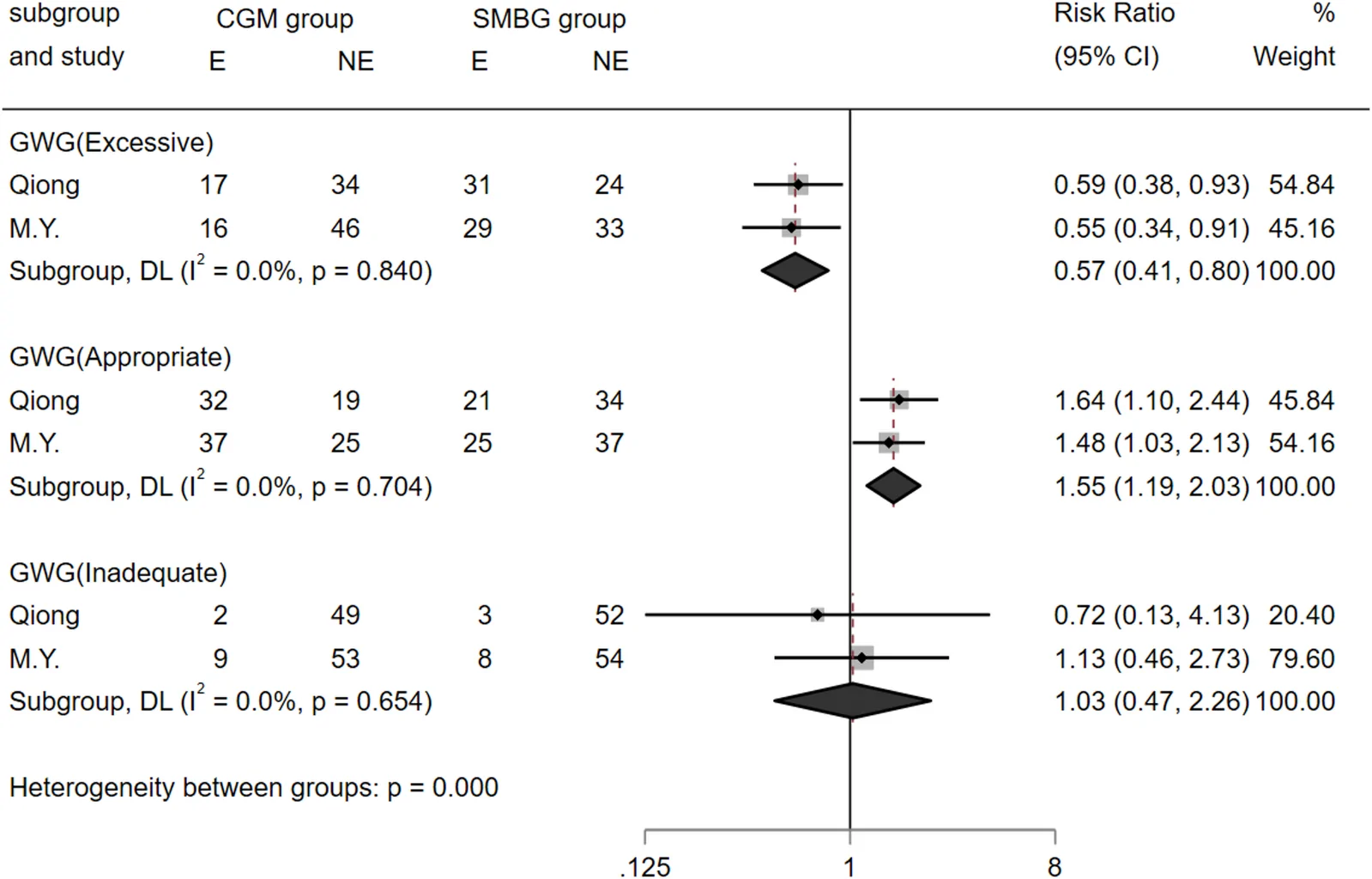
Gestational weight gain in the CGM and SMBG groups. CGM: Continuous Glucose Monitoring; SMBG: Self-monitoring of blood glucose; 95% CI: 95% Confidence Interval; Risk Ratio: RR. Gestational weight gain (GWG) outcomes encompass subgroups categorized as excessive gestational weight gain, appropriate gestational weight gain, and inadequate gestational weight gain

A total of 2 studies from 4 randomized controlled trials involving 425 participants assessed the effectiveness of CGM compared with SMBG with respect to three categories of GWG, namely, excessive, appropriate, and inadequate GWG [37]. The results of the study showed that CGM was significantly associated with a decrease in the number of GWG-excessive and an increase in the number of GWG-appropriate (RR = 0.57; 95% CI: 0.41, 0.80; I² = 0.0%; *P* = 0.001), (RR = 1.55; 95% CI: 1.19, 2.03; I² = 0.0%; *P* = 0.001). However, the GWG-Inadequate outcome was not statistically significant (RR = 1.03; 95% CI = 0.47, 2.26; I² = 0.0%; *P* = 0.948). See Fig. 2 for details.

### Glucose control

Among the 4 randomized controlled trials involving 425 participants. Three studies compared the effects of CGM on HbA1c with those of SMBG [37, 39, 40]. Two studies reported glucose-lowering drug doses(SMD = 0.04; 95% CI=-0.57, 0.65; I2 = 81.6%; *P* = 0.910). The results revealed no statistically significant difference in HbA1c or the number of people treated with hypoglycemic drugs (SMD=-0.36; 95% CI=-0.71, 0.00; I^2^ = 65.1%; *P* = 0.051), (RR = 1.66; 95% CI = 0.98, 2.84; I^2^ = 43.2%; *P* = 0.061). See Additional file 2 for details.

### Secondary outcome

#### Maternal and neonatal outcomes

Among pregnant women, cesarean section outcomes were evaluated in three studies. The pooled results indicated no statistically significant difference (RR = 0.91; 95% CI: 0.75, 1.12; I²=0.0%; *P* = 0.389). In terms of neonatal outcomes, newborns with a birth weight greater than 4000 g were classified as macrosomia, and four trials reported this outcome (RR = 0.81; 95% CI = 0.35, 1.86; I² = 0.0%; *P* = 0.620) [44]. Hyperbilirubinemia was reported in three trials(RR = 1.01; 95% CI = 0.56, 1.83; I² = 0.0%; *P* = 0.962). Three trials reported neonatal NICU admission (RR = 0.97; 95% CI = 0.65, 1.44; I² = 0.0%; *P* = 0.863). Four trials reported neonatal hypoglycemia (RR = 1.10; 95% CI = 0.60, 2.02; I² = 0.0%; *P* = 0.748). Two trials provided separate reports on LGA (RR = 0.70; 95% CI = 0.46, 1.07; I² = 0.0%; *P* = 0.100) and SGA (RR = 0.70; 95% CI = 0.20, 2.46; I² = 0.0%; *P* = 0.579). Two studies reported Apgar scores (SMD = -0.16; 95% CI = -0.45, 0.14; I² = 0.0%; *P* = 0.295). Four studies reported neonatal birth weight (SMD = -0.31; 95% CI = -0.50, -0.11; I² = 0.0%; *P* = 0.002). See Additional file 2 for details.

### Subgroup

To investigate the differential effects of various CGM modalities, we conducted separate subgroup analyses for r-CGM and rt-CGM, comparing their respective differences in outcomes for patients monitored with CGM and SMBG.

In terms of r-CGM, the effects of r-CGM on HbA1c, hyperbilirubinemia, macrosomia, neonatal NICU, and neonatal hypoglycemic outcomes were not significantly different from those of SMBG, respectively. The effect of r-CGM on the number of people treated with hypoglycemic drugs(RR = 2.10; 95%CI = 1.16, 3.80; I^2^ = 19.1%; *P* = 0.004) as well as birth weight outcome(SMD=-0.30; 95%=-0.54, -0.06; I^2^ = 8.6%; *P* = 0.009) was significant different from those of SMBG.

**Fig. 3:**
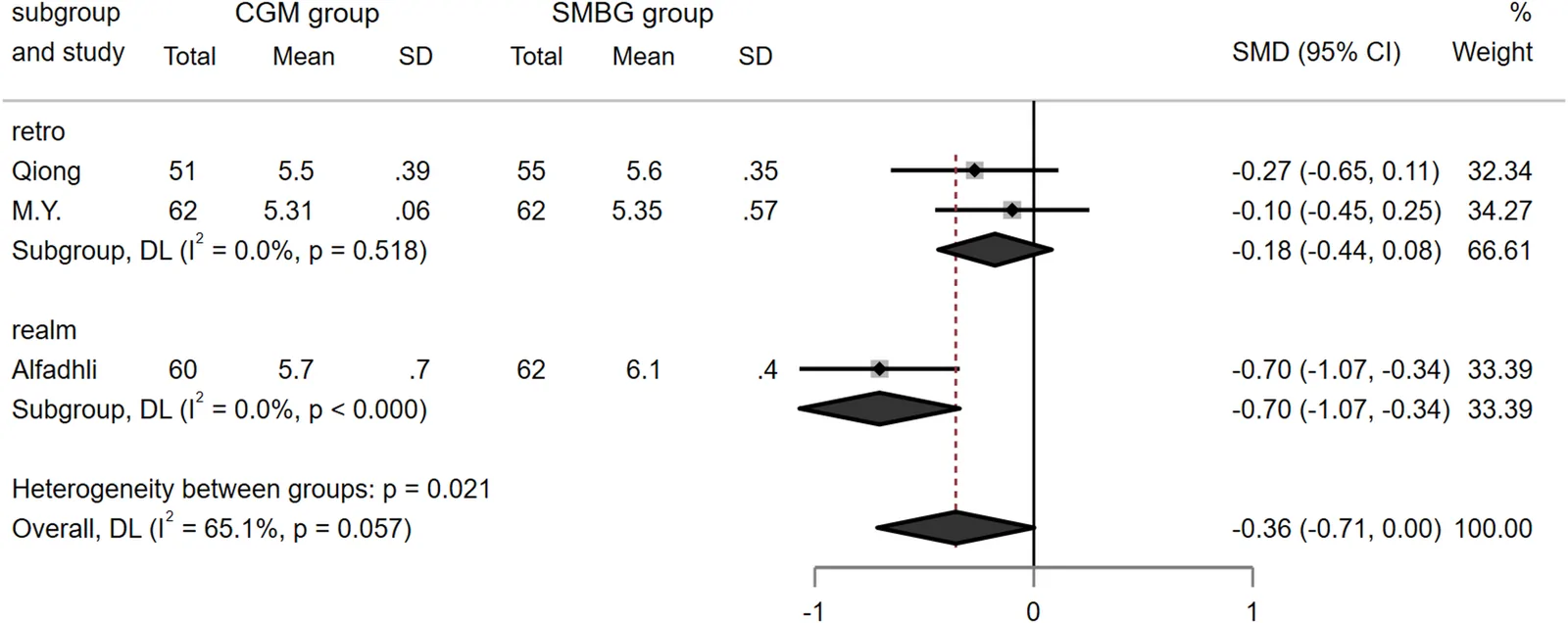
Forest plot of HbA1c in retrospective (retro) and real-time (realm) CGM subgroups versus SMBG. retro: retrospective continuous glucose monitoring; realm: real-time continuous glucose monitoring; SMBG：Self-monitoring of blood glucose; 95% CI: 95% Confidence Interval ;SMD: Standardized Mean Difference. HbA1c: haemoglobin A1c

**Fig. 4:**
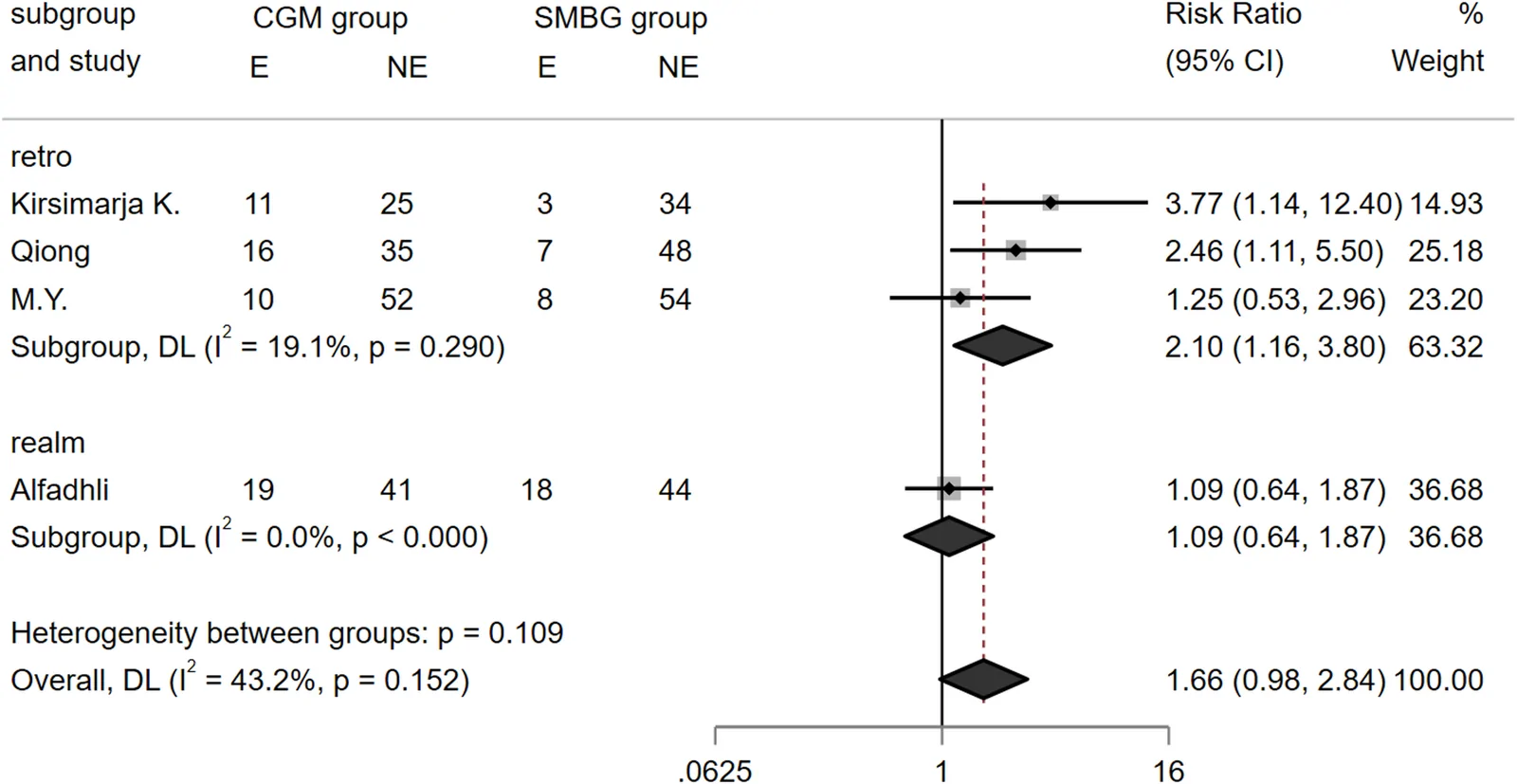
Forest plot of hypoglycemic drug use in retrospective (retro) and real-time (realm) CGM subgroups versus SMBG. retro: retrospective continuous glucose monitoring; realm: real-time continuous glucose monitoring; SMBG：Self-monitoring of blood glucose; 95% CI: 95% Confidence Interval ; Risk Ratio: RR

In the terms of rt-CGM, rt-CGM reduced HbA1c values compared to SMBG(SMD=-0.70; 95%CI=-1.07, -0.34; I^2^ = 0.0%; *P* < 0.001), and the effects of rt-CGM on hyperbilirubinemia, macrosomia, neonatal NICUs, neonatal hypoglycemia, the number of people treated with hypoglycemic drugs, and newborn weight outcomes were not significantly different from those of SMBG. Subgroup analyses of glycemic outcomes and perinatal outcomes are summarized in Additional file 3. See Figs. 3 and 4 and Additional file 2 for details.

### Sensitivity analyses

To evaluate the robustness of the results, we conducted sensitivity analyses and substituted different effect models for the primary outcomes. The sensitivity analysis revealed that the outcomes for GWG-Excessive (ES = 0.57, 95% CI = 0.41, 0.80), GWG-Appropriate (ES = 1.55, 95% CI = 1.19, 2.03), and GWG-Inadequate (ES = 1.03, 95% CI = 0.47, 2.03) demonstrated substantial robustness. The outcomes related to GWG demonstrated substantial consistency between the random effects and fixed effects models. However, model substitution resulted in divergent findings regarding the number of people treated with hypoglycemic drugs. Sensitivity analysis, conducted through a leave-one-out approach, identified the study by Alfadli et al. as the primary contributor to the observed inconsistencies (ES = 1.66, 95% CI = 0.98, 2.84). Furthermore, subgroup analyses stratified by the method of CGM monitoring revealed that variations in CGM protocols could be a crucial factor contributing to the inconsistency in model fit. Notably, the heterogeneity of the model was significantly diminished upon the exclusion of the study by Alfadli et al. However, in the HbA1c outcome, significance was observed after a fixed-effects model was employed (*P* < 0.05), whereas the random-effects model did not yield significant results. Additionally, the sensitivity analyses using the leave-one-out method demonstrated a lack of robustness. This discrepancy may be attributed to the small overall sample size and the limited number of studies included. Future research should aim to increase the sample size and investigate the influence of different CGM monitoring modalities on the heterogeneity of outcomes to strengthen the robustness and generalizability of the perinatal results. See Additional file 2 for details.

### Risk of bias assessment

**Fig. 5:**
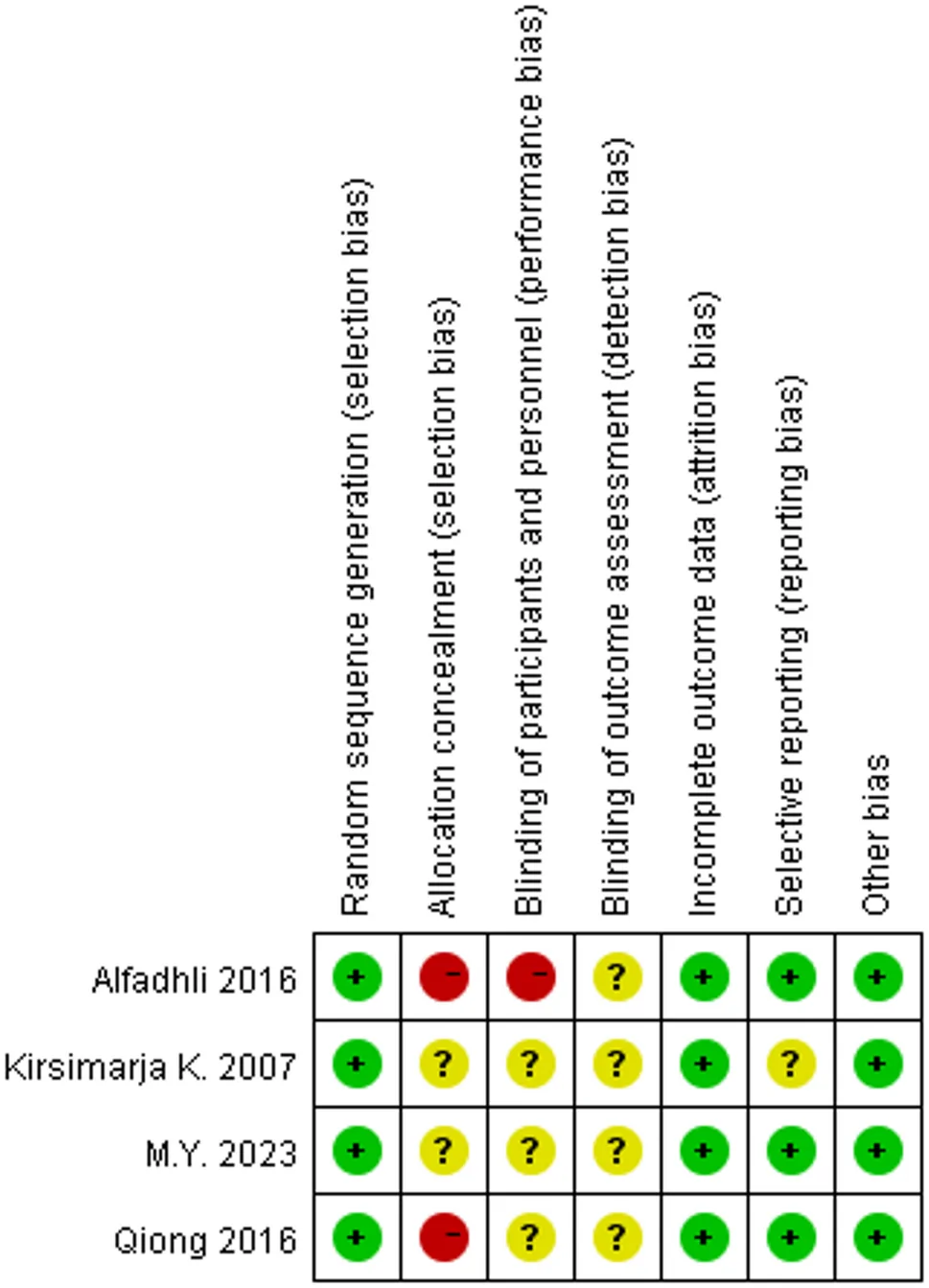
Risk of bias summary

Literature quality was evaluated via the Cochrane Handbook on Risk of Bias for risk assessment of the level of evidence [45]. The process of randomized sequence generation was reported in all 4 studies. Two studies were open RCTs, and two studies did not publish blinding of participants and personnel. All four studies provided plausible explanations for the incomplete outcome data. All studies reported findings consistent with the intended objectives. The combined assessment of the included literature indicated that two studies were at high risk, and two studies presented some risk. All studies followed preregistered protocols for the analysis and reporting of results. Thus, the overall methodological quality was moderate, and larger sample sizes and carefully conducted randomized controlled trials are needed in the future. See Fig. 5 for details.

## Discussion

### Principal findings

In this systematic review and meta-analysis, data from four studies involving 425 noninsulin**-**treated GDM women who underwent either CGM or SMBG interventions were pooled [37–40]. The findings demonstrated that CGM had a significantly stronger association than SMBG in preventing GWG-Excessive and maintaining GWG-Appropriate in women with GDM. However, no significant difference was observed between CGM and SMBG in preventing inadequate GWG. This may be attributed to the limited number of women in the study population who experienced inadequate GWG, potentially restricting the statistical power to detect an intervention effect. At the level of glycemic control, compared with SMBG, r-CGM showed comparable effectiveness in terms of HbA1c outcomes, whereas rt-CGM appeared to offer a superior improvement in HbA1c levels. However, the findings related to rt-CGM are limited by the inclusion of only a single study. Moreover, compared with SMBG, r-CGM was associated with a 110% increased risk of requiring glucose-lowering medication. In contrast, rt-CGM did not significantly differ from SMBG in terms of the number of people treated with hypoglycemic drugs, although this result is constrained by the limited number of studies available.

### Comparison with previous studies

Excessive gestational weight gain and inadequate GWG are both strongly associated with adverse outcomes for both GDM mothers and their newborns [46–48]. Effective management of GWG in GDM pregnancies may involve interventions such as dietary modifications, SMBG, and frequent follow-up visits [49]. SMBG is a crucial component of weight management, and fluctuations in blood glucose levels during pregnancy may be linked to excessive-GWG. This is because excessive weight gain can lead to abnormal fat accumulation, which may impair insulin sensitivity and result in β-cell dysfunction. Furthermore, pregnant women with GDM inherently exhibit chronic, progressive insulin resistance, a condition that predisposes them to glycemic fluctuations [50]. BGM methods, such as CGM and SMBG, facilitate the timely detection of glucose fluctuations and enable pregnant women to make necessary adjustments to their dietary and physical activity patterns. Therefore, blood glucose monitoring plays a critical role in regulating blood glucose levels, which can, in turn, indirectly contribute to improved weight management in pregnant women with GDM [51]. CGM has the potential to overcome the limitations of SMBG, particularly the issue of temporal gaps in measurements, which may result in missing blood glucose peaks. By providing continuous and comprehensive data on blood glucose fluctuations, CGM offers a more accurate representation of glycemic patterns, thereby suggesting its potential advantage over SMBG in enhancing weight management in patients [11].

Most previous systematic reviews have focused predominantly on overall GWG in pregnant women, and the effects of CGM versus SMBG on GWG outcomes remain controversial [26–28]. For example, Chang et al. demonstrated that, compared with SMBG, CGM did not significantly reduce GWG. Their study population included individuals with T1DM or T2DM [27]; however, the analysis was not stratified according to different population subgroups. In contrast, a study by García-Moreno et al. specifically focused on patients with GDM and demonstrated that CGM significantly reduced GWG compared with BGM [26]. Owing to the observed inconsistencies in GWG outcomes, our meta-analysis investigated varying levels of GWG and evaluated the effectiveness of CGM versus SMBG in impacting GWG outcomes. The present study is the first to perform a more nuanced analysis of GWG levels in pregnant women with GDM, categorizing them into excessive, appropriate, and inadequate weight gain. Heterogeneity was negligible (0%) across all GWG categories, and the findings demonstrated robustness in sensitivity analyses. This study offers more accurate evidence to inform targeted weight management strategies across various levels of GDM, particularly considering the distinct patterns of weight gain.

Secondly, although neonatal birth weight is a secondary outcome, we believe it is necessary to discuss it. r-CGM enables clinicians to comprehensively assess the historical blood glucose levels of diabetic patients, thereby influencing their dietary and exercise habits, which may indirectly affect maternal weight management and neonatal birth weight [52]. Previous studies have suggested that r-CGM is beneficial in reducing the risk of macrosomia in pregnant women with diabetes [53]. However, our findings on macrosomia did not demonstrate a significant effect. We speculate that this discrepancy may be attributed to the relatively small number of macrosomia cases included in our study and the broad definition of macrosomia. Therefore, future large-scale RCTs are warranted to further investigate this issue.

In addition, with regard to glucose control outcomes, owing to inconsistencies between the sensitivity analyses and the primary analyses, we conducted subgroup analyses to further explore the effectiveness of different types of CGM, specifically r-CGM versus rt-CGM, in comparison to SMBG. These analyses focused on glucose control as well as the necessity for a number of people treated with hypoglycemic drugs in patients with GDM. With respect to HbA1c values, our findings diverged from those reported by Chang et al. who concluded that r-CGM was superior to rt-CGM in terms of glucose control in pregnant women [27]. In contrast, our results align closely with those of Ida et al. [54, 55]. The discrepancies in findings across studies may stem from several factors, including differences in the types of diabetes mellitus, variations in the study populations, and the inclusion of different control groups. Our findings regarding the number of people treated with hypoglycemic drugs align with those reported by García-Moreno et al. However, their study did not perform a subgroup analysis on the basis of the specific modality of the CGM intervention [26]. Notably, the disparity in outcomes between r-CGM and rt-CGM became more evident upon further subgroup analysis.

We believe that r-CGM can be used to observe fluctuations in blood glucose over time by reviewing blood glucose data from previous days. In addition, rt-CGM offers real-time feedback by continuously monitoring glucose levels [56]. This difference in outcomes may stem from the inability of r-CGM to detect glucose fluctuations in a timely manner, which could lead to a preference for pharmacological interventions to manage blood glucose [55]. Conversely, real-time monitoring of rt-CGM allows prompt adjustments to avoid hyperglycemia, thereby reducing the number of people treated with hypoglycemic drugs [57]. For patients with GDM, lifestyle interventions such as dietary modification and physical activity are the primary treatment approaches. However, when these interventions fail to achieve optimal glycemic control, glucose-lowering medications become necessary. rt-CGM allows continuous monitoring of blood glucose fluctuations throughout the day and night via glucose sensors, capturing both hyperglycemic and hypoglycemic events [58]. By utilizing rt-CGM, patients can track glucose trends in real time and make timely adjustments to their diet and exercise regimens [58, 59]. Previous limited studies have demonstrated that rt-CGM facilitates weight loss, reduces the intake of high glycemic index foods, and promotes dietary fiber consumption in patients with T2DM through lifestyle modifications [60]. Although these findings are based on the T2DM population, they suggest that rt-CGM may offer similar benefits for patients with GDM. By optimizing glycemic management, rt-CGM has the potential to reduce the proportion of GDM patients requiring glucose-lowering medications.

However, due to the heterogeneity of the studies and the limited number of studies, the findings regarding glycemic control and number of people treated with hypoglycemic drugs need to be confirmed by larger RCTs.

### Strengths and limitations

This meta-analysis presents several notable strengths. First, we conducted a comprehensive search of five major English-language electronic databases, utilizing both MeSH terms and free-text keywords to mitigate publication bias. Second, we preregistered the study on PROSPERO in accordance with PRISMA guidelines, employed a robust three-step search strategy, and tracked citations to ensure a thorough literature scope. Third, the study population consisted of pregnant women in their second and third trimesters, with a consistent frequency of daily glucose monitoring and participation in overall glucose monitoring. Fourth, the overall heterogeneity of the included studies was low, indicating good reliability and scientific interpretability. Fifth, factors such as lifestyle interventions have minimal influence on the validity of comparing the outcomes of CGM and SMBG. However, this study has several limitations. First, the sample sizes of the included studies were generally small, with a scarcity of large-scale studies. Second, relevant non-English literature was excluded from the analysis. Third, the limited number of studies included in this analysis restricts the generalizability and robustness of our findings. Fourth, Short-term use of CGM may not fully capture the glucose patterns across the entire duration of pregnancy. Fifth, only one included study utilized rt-CGM, which limited our ability to perform subgroup analyses and may affect the generalizability of the findings related to rt-CGM. Sixth, the absence of stratified data distinguishing between oral and injectable hypoglycemic agents limits the ability to assess their differential effects on clinical outcomes.

### Research implications

Although existing studies have explored the application of CGM, further investigations are warranted in patients with GDM [26–28]. The evidence on perinatal outcomes associated with GDM remains limited and inconsistent [26, 27]. Moreover, prior systematic reviews and meta-analyses have focused primarily on comparisons between CGM and BGM, which differ significantly from the SMBG utilized in this study, as SMBG places greater emphasis on patient-driven monitoring [26]. The comparative effects of CGM and SMBG in the GDM population remain inconclusive. Additionally, earlier studies conducted subgroup analyses of CGM, often including diverse populations with diabetes [27]. These analyses suggest that retrospective CGM may outperform real-time CGM in terms of glycemic control [27]. Therefore, this study aims to further investigate the effects of CGM and SMBG on glycemic control and perinatal outcomes in pregnant women with GDM in home settings and to perform subgroup analyses of CGM modalities to clarify their clinical implications.

## Conclusions

CGM may be more effective than SMBG in preventing excessive weight gain and maintaining appropriate gestational weight in women with GDM. Moreover, rt-CGM appears to offer superior glycemic control compared with SMBG. r-CGM may be more effective in reducing neonatal birth weight. Future studies should investigate the impact of short-term vs. long-term CGM use on maternal and neonatal outcomes.

## Supplementary Information

Supplementary Material 1.

Supplementary Material 2.

Supplementary Material 3.

## Data Availability

The datasets used in this manuscript is available from the corresponding author on a reasonable request.
