# Supplementary material for: A systematic review and meta-analysis of the effects of continuous glucose monitoring compared to self-monitoring of blood glucose on glycemic control and maternal and infant outcomes in women with gestational diabetes mellitus
Source: BMC Pregnancy Childbirth. 2026 Aug 8;26:1272. doi: 10.1186/s12884-026-09769-9 (PMC13733083; doi:10.1186/s12884-026-09769-9)
Supplement: Supplementary file 1 [file 12884_2026_9769_MOESM1_ESM.docx]

| **#** | **AppendiX(A.1) Searches (PubMed)** | **outcomes** |
| --- | --- | --- |
| 1 | Randomized Controlled Trial" [Publication Type] Sort by: Most Recent |  |
| 2 | ("Blood Glucose Self-Monitoring"[Mesh] OR ((((((((((((((((((("blood glucose self monitoring"[Title/Abstract]) OR ("glucose, blood, self-monitoring"[Title/Abstract])) OR ("self-monitoring, blood glucose"[Title/Abstract])) OR ("blood glucose self-monitorings"[Title/Abstract])) OR ("glucose self-monitoring, blood"[Title/Abstract])) OR ("glucose self-monitorings, blood"[Title/Abstract])) OR ("self monitoring, blood glucose"[Title/Abstract])) OR ("self-monitorings, blood glucose"[Title/Abstract])) OR ("monitoring, home blood glucose"[Title/Abstract])) OR ("blood sugar self-monitoring"[Title/Abstract])) OR ("blood sugar self monitoring"[Title/Abstract])) OR ("blood sugar self-monitorings"[Title/Abstract])) OR ("self-monitoring, blood sugar"[Title/Abstract])) OR ("self-monitorings, blood sugar"[Title/Abstract])) OR ("sugar self-monitoring, blood"[Title/Abstract])) OR ("sugar self-monitorings, blood"[Title/Abstract])) OR ("home blood glucose monitoring"[Title/Abstract])) OR ("glucose, blood, self monitoring"[Title/Abstract])) OR ("smbg"[Title/Abstract])))) OR (("Continuous Glucose Monitoring"[Mesh]) OR ((((((((((("Glucose Monitoring, Continuous"[Title/Abstract])) OR ("Monitoring, Continuous Glucose"[Title/Abstract])) OR ("Monitorings, Continuous Glucose"[Title/Abstract])) OR ("Continuous Glucose Monitoring Device"[Title/Abstract])) OR ("CGM Device"[Title/Abstract])) OR ("CGM Devices"[Title/Abstract])) OR ("Device, CGM"[Title/Abstract])) OR ("Devices, CGM"[Title/Abstract])) OR ("CGM"[Title/Abstract])) OR ("CGMS"[Title/Abstract]))) | 14426 |
| 3 | ((("Diabetes, Gestational"[Mesh]) OR (((((("Diabetes, Pregnancy-Induced"[Title/Abstract]) OR ("Diabetes, Pregnancy Induced"[Title/Abstract])) OR ("Pregnancy-Induced Diabetes"[Title/Abstract])) OR ("Gestational Diabetes"[Title/Abstract])) OR ("Diabetes Mellitus, Gestational"[Title/Abstract])) OR ("Gestational Diabetes Mellitus"[Title/Abstract]))) OR ((((((("Pregnancy in Diabetics"[Title/Abstract]) OR ("Diabetes, Gestational"[Title/Abstract])) OR ("GDM"[Title/Abstract])) OR (("diabet*"[Title/Abstract]) AND ("pregnan*"[Title/Abstract]))) OR (("pregnan*"[Title/Abstract]) AND ("abnormal oral glucose"[Title/Abstract]))) OR (("abnormal glucose"[Title/Abstract]) AND ("pregnan*"[Title/Abstract]))) OR (("glucose intoleran*"[Title/Abstract]) AND ("pregnan*"[Title/Abstract])))) OR ((((((((("gestational hyperglycemia "[Title/Abstract]) OR ("gestational diabete*"[Title/Abstract])) OR ("gestational glucose "[Title/Abstract])) OR ("pregnancy hyperglycemia"[Title/Abstract])) OR ("pregnancy diabete* "[Title/Abstract])) OR ("pregnancy glucose "[Title/Abstract])) OR ("maternal hyperglycemia"[Title/Abstract])) OR ("maternal diabete* "[Title/Abstract])) OR ("maternal glucose"[Title/Abstract])) | 47333 |
| 4 | (((((((((((((((((((((((((("*Gastrointestinal Hemorrhage"[Title/Abstract]) OR ("Variceal bleed"[Title/Abstract])) OR ((("Pregnancy Complications"[Title/Abstract]) OR ("*Postoperative Hemorrhage "[Title/Abstract])) OR ("Hemorrhage "[Title/Abstract]))) OR ("Hematemesis "[Title/Abstract])) OR (("Abruptio Placentae "[Title/Abstract]) OR ("Abruptio"[Title/Abstract]))) OR (("*Hypertension, Pregnancy-Induced"[Title/Abstract]) OR ("Hypertension "[Title/Abstract]))) OR ((("Preeclampsia "[Title/Abstract]) OR ("Pre-Eclampsia"[Title/Abstract])))) OR (("Thrombocytopenia, Neonatal Alloimmune "[Title/Abstract]) OR ("Thrombocytopenia "[Title/Abstract]))) OR ("Anemia "[Title/Abstract])) OR (("Blood transfusion "[Title/Abstract]) OR ("Blood Transfusion"[Title/Abstract]))) OR (("Platelet transfusion "[Title/Abstract]) OR ("*Platelet Transfusion"[Title/Abstract]))) OR (("Decompensation"[Title/Abstract]) OR ("*Jaundice, Neonatal "[Title/Abstract]))) OR ((("Ascites "[Title/Abstract]) OR ("Sepsis "[Title/Abstract])) OR ("sepsis"[Title/Abstract]))) OR (("Maternal death "[Title/Abstract]) OR ("Maternal Death"[Title/Abstract]))) OR (("Maternal mortality "[Title/Abstract]) OR ("Maternal Mortality"[Title/Abstract]))) OR ("Fetal complications"[Title/Abstract])) OR (((("*Abortion, Habitual "[Title/Abstract]) OR ("*Abortion, Spontaneous "[Title/Abstract])) OR ("*Abortion, Induced "[Title/Abstract])))) OR (("Low birth weight "[Title/Abstract]) OR ("Infant, Low Birth Weight"[Title/Abstract]))) OR ((("Cesarean Section "[Title/Abstract]) OR ("Mode of Delivery "[Title/Abstract])) OR ("Delivery, Obstetric"[Title/Abstract]))) OR ((("Premature Birth "[Title/Abstract]) OR ("Preterm delivery "[Title/Abstract])) OR ("*Obstetric Labor, Premature"[Title/Abstract]))) OR (("Intrauterine growth restriction "[Title/Abstract]) OR ("Fetal Growth Retardation"[Title/Abstract]))) OR (("small for gestational age"[Title/Abstract]))) OR ((("*Intensive Care Units, Neonatal "[Title/Abstract]) OR ("NICU admission"[Title/Abstract])) OR ("neonatal intensive care unit"[Title/Abstract]))) OR (("Neonatal death "[Title/Abstract]) OR ("Perinatal Death"[Title/Abstract]))) OR ((((((("fetal-outcom*"[Title/Abstract]) OR ("fetus outcome*"[Title/Abstract])) OR ("obstetric* outcome*"[Title/Abstract])) OR ("maternal-outcom*"[Title/Abstract])) OR ("neonatal-outcom*"[Title/Abstract])) OR ("perinatal-outcom*"[Title/Abstract])) OR ("Maternal Outcomes"[Title/Abstract]))) OR ((((((((((((((("Spontaneous miscarriage"[Title/Abstract]) OR ("Pregnancy loss"[Title/Abstract])) OR ("Antepartum hemorrhage"[Title/Abstract])) OR ("Post-partum hemorrhage"[Title/Abstract])) OR ("Induction of labour"[Title/Abstract])) OR ("Instrumental birth"[Title/Abstract])) OR ("Caesarean section"[Title/Abstract])) OR ("Shoulder dystocia"[Title/Abstract])) OR ("Eclampsia"[Title/Abstract])) OR ("Placenta previa "[Title/Abstract])) OR ("Placental abruption"[Title/Abstract])) OR ("Premature rupture of membrane"[Title/Abstract])) OR ("Infection"[Title/Abstract])) OR ("Amniotic embolism"[Title/Abstract])) OR ("Hyperemesis gravidarum"[Title/Abstract]))) OR ((((((((((((((((((((((("mortality"[Title/Abstract]) OR ("death"[Title/Abstract])) OR ("stillbirth"[Title/Abstract])) OR ("congenital"[Title/Abstract])) OR ("malformation"[Title/Abstract])) OR ("anomaly"[Title/Abstract])) OR ("impair* "[Title/Abstract])) OR ("disabilit*"[Title/Abstract])) OR ("macrosomia"[Title/Abstract])) OR ("lbw"[Title/Abstract])) OR ("large for gestational age"[Title/Abstract])) OR ("LGA"[Title/Abstract])) OR ("SGA"[Title/Abstract])) OR ("neonatal hypoglyc*"[Title/Abstract])) OR ("neonatal hyperbili*"[Title/Abstract])) OR ("jaundice"[Title/Abstract])) OR ("respiratory distress syndrome"[Title/Abstract])) OR ("RDS"[Title/Abstract])) OR ("hyaline membrane disease"[Title/Abstract])) OR ("HMD"[Title/Abstract])) OR ("Apgar"[Title/Abstract])) OR ("asphyxia"[Title/Abstract])) OR ("apnoea"[Title/Abstract])) | 5374841 |
| 4 | #1 AND #2 AND #3 AND #4 | 44 |

| **#** | **Appendix(A.2) Searches (Medline from Web of science)** | **Outcome** |
| --- | --- | --- |
| 11 | (((((((((((((((((((((((((((((TS=("blood glucose self monitoring")) OR TS=("glucose, blood, self-monitoring")) OR TS=("self-monitoring, blood glucose")) OR TS=("blood glucose self-monitorings")) OR TS=("glucose self-monitoring, blood")) OR TS=("glucose self-monitorings, blood")) OR TS=("self monitoring, blood glucose")) OR TS=("self-monitorings, blood glucose")) OR TS=("monitoring, home blood glucose")) OR TS=("blood sugar self-monitoring")) OR TS=("blood sugar self monitoring")) OR TS=("blood sugar self-monitorings")) OR TS=("self-monitoring, blood sugar")) OR TS=("self-monitorings, blood sugar")) OR TS=("sugar self-monitoring, blood")) OR TS=("sugar self-monitorings, blood")) OR TS=("home blood glucose monitoring")) OR TS=("glucose, blood, self monitoring")) OR TS=("smbg")) OR TS=("Continuous Glucose Monitoring")) OR TS=("Glucose Monitoring, Continuous")) OR TS=("Monitoring, Continuous Glucose")) OR TS=("Monitorings, Continuous Glucose")) OR TS=("Continuous Glucose Monitoring Device")) OR TS=("CGM Device")) OR TS=("CGM Devices")) OR TS=("Device, CGM")) OR TS=("Devices, CGM")) OR TS=("CGM")) OR TS=("CGMS") | 15869 |
| 13 | ((((((TS=("Diabetes, Gestational")) OR TS=("Diabetes, Pregnancy-Induced")) OR TS=("Diabetes, Pregnancy Induced")) OR TS=("Pregnancy-Induced Diabetes")) OR TS=("Gestational Diabetes")) OR TS=("Diabetes Mellitus, Gestational")) OR TS=("Gestational Diabetes Mellitus") | 25736 |
| 14 | ((((((((TS=("gestational hyperglycemia ")) OR TS=("gestational diabete* ")) OR TS=("gestational glucose ")) OR TS=("pregnancy hyperglycemia")) OR TS=("pregnancy diabete* ")) OR TS=("pregnancy glucose ")) OR TS=("maternal hyperglycemia")) OR TS=("maternal diabete* ")) OR TS=("maternal glucose") | 26472 |
| 15 | ((TS=("Pregnancy in Diabetics" )) OR TS=("Diabetes, Gestational")) OR TS=("GDM") | 29867 |
| 16 | (TS=("diabet*")) AND TS=("pregnan*") | 51030 |
| 17 | (TS=("abnormal oral glucose")) AND TS=("pregnan*") | 67 |
| 18 | (TS=("abnormal glucose")) AND TS=("pregnan*") | 586 |
| 19 | (TS=("glucose intoleran*")) AND TS=("pregnan*") | 2337 |
| 20 | #13 OR #14 OR #15 OR #16 OR #17 OR #18 OR #19 | 54393 |
| 21 | ((((((((((((((((((((((TS=("mortality")) OR TS=("death")) OR TS=("stillbirth")) OR TS=("congenital")) OR TS=("malformation")) OR TS=("anomaly")) OR TS=("impair* ")) OR TS=("disabilit*")) OR TS=("macrosomia")) OR TS=("lbw")) OR TS=("large for gestational age")) OR TS=("LGA")) OR TS=("SGA")) OR TS=("neonatal hypoglyc*")) OR TS=("neonatal hyperbili*")) OR TS=("jaundice")) OR TS=("respiratory distress syndrome")) OR TS=("RDS")) OR TS=("hyaline membrane disease")) OR TS=("HMD")) OR TS=("Apgar")) OR TS=("asphyxia")) OR TS=("apnoea") | 3902857 |
| 22 | ((((((((((((((TS=("Spontaneous miscarriage")) OR TS=("Pregnancy loss")) OR TS=("Antepartum hemorrhage")) OR TS=("Post-partum hemorrhage")) OR TS=("Induction of labour")) OR TS=("Instrumental birth")) OR TS=("Caesarean section")) OR TS=("Shoulder dystocia")) OR TS=("Eclampsia")) OR TS=("Placenta previa ")) OR TS=("Placental abruption")) OR TS=("Premature rupture of membrane")) OR TS=("Infection")) OR TS=("Amniotic embolism")) OR TS=("Hyperemesis gravidarum") | 1615509 |
| 23 | ((((((TS=("fetal-outcom*")) OR TS=("fetus outcome*")) OR TS=("obstetric* outcome*")) OR TS=("maternal-outcom*")) OR TS=("neonatal-outcom*")) OR TS=("perinatal-outcom*")) OR TS=("Maternal Outcomes") | 38430 |
| 24 | ((TS=("*Gastrointestinal Hemorrhage ")) OR TS=("Variceal bleed")) OR TS=("Pregnancy Complications") | 232242 |
| 25 | ((TS=("*Postoperative Hemorrhage ")) OR TS=("Hemorrhage ")) OR TS=("Hematemesis ") | 340130 |
| 26 | (((TS=("Abruptio Placentae ")) OR TS=("Abruptio")) OR TS=("Hypertension ")) OR TS=("*Hypertension, Pregnancy-Induced") | 591804 |
| 27 | (((TS=("Preeclampsia ")) OR TS=("Pre-Eclampsia")) OR TS=("Thrombocytopenia, Neonatal Alloimmune ")) OR TS=("Thrombocytopenia ") | 126072 |
| 28 | ((TS=("Anemia ")) OR TS=("Blood transfusion ")) OR TS=("Blood Transfusion") | 291418 |
| 29 | ((TS=("Platelet transfusion ")) OR TS=("*Platelet Transfusion")) OR TS=("Decompensation") | 24629 |
| 30 | (((TS=("*Jaundice, Neonatal ")) OR TS=("Ascites ")) OR TS=("Sepsis ")) OR TS=("sepsis") | 216392 |
| 31 | (((TS=("Maternal death ")) OR TS=("Maternal Death")) OR TS=("Maternal mortality ")) OR TS=("Maternal Mortality") | 22301 |
| 32 | (((TS=("Fetal complications")) OR TS=("*Abortion, Habitual ")) OR TS=("*Abortion, Spontaneous ")) OR TS=("*Abortion, Induced ") | 60253 |
| 33 | (TS=("Low birth weight ")) OR TS=("Infant, Low Birth Weight") | 49494 |
| 34 | (TS=("Mode of Delivery ")) OR TS=("Delivery, Obstetric") | 40912 |
| 35 | (((TS=("Cesarean section")) OR TS=("Premature Birth ")) OR TS=("Preterm delivery ")) OR TS=("*Obstetric Labor, Premature") | 110442 |
| 36 | ((TS=("Intrauterine growth restriction ")) OR TS=("Fetal Growth Retardation")) OR TS=("small for gestational age") | 36966 |
| 37 | ((TS=("*Intensive Care Units, Neonatal ")) OR TS=("NICU admission")) OR TS=("neonatal intensive care unit") | 31593 |
| 38 | (TS=("Neonatal death ")) OR TS=("Perinatal Death") | 10694 |
| 39 | #21 OR #22 OR #23 OR #24 OR #25 OR #26 OR #27 OR #28 OR #29 OR #30 OR #31 OR #32 OR #33 OR #34 OR #35 OR #36 OR #37 OR #38 | 6454982 |
| 40 | ((TS=(randomized controlled trial)) OR TS=(randomized)) OR TS=(placebo) | 1027349 |
| 41 | #11 AND #20 AND #39 AND #40 | 84 |

| **#** | **Appendix (A.3) Searches（Web of Science Core database）** | **Outcome** |
| --- | --- | --- |
| 11 | (((((((((((((((((((((((((((((TS=("blood glucose self monitoring")) OR TS=("glucose, blood, self-monitoring")) OR TS=("self-monitoring, blood glucose")) OR TS=("blood glucose self-monitorings")) OR TS=("glucose self-monitoring, blood")) OR TS=("glucose self-monitorings, blood")) OR TS=("self monitoring, blood glucose")) OR TS=("self-monitorings, blood glucose")) OR TS=("monitoring, home blood glucose")) OR TS=("blood sugar self-monitoring")) OR TS=("blood sugar self monitoring")) OR TS=("blood sugar self-monitorings")) OR TS=("self-monitoring, blood sugar")) OR TS=("self-monitorings, blood sugar")) OR TS=("sugar self-monitoring, blood")) OR TS=("sugar self-monitorings, blood")) OR TS=("home blood glucose monitoring")) OR TS=("glucose, blood, self monitoring")) OR TS=("smbg")) OR TS=("Continuous Glucose Monitoring")) OR TS=("Glucose Monitoring, Continuous")) OR TS=("Monitoring, Continuous Glucose")) OR TS=("Monitorings, Continuous Glucose")) OR TS=("Continuous Glucose Monitoring Device")) OR TS=("CGM Device")) OR TS=("CGM Devices")) OR TS=("Device, CGM")) OR TS=("Devices, CGM")) OR TS=("CGM")) OR TS=("CGMS") | 20168 |
| 13 | ((((((TS=("Diabetes, Gestational")) OR TS=("Diabetes, Pregnancy-Induced")) OR TS=("Diabetes, Pregnancy Induced")) OR TS=("Pregnancy-Induced Diabetes")) OR TS=("Gestational Diabetes")) OR TS=("Diabetes Mellitus, Gestational")) OR TS=("Gestational Diabetes Mellitus") | 32823 |
| 14 | ((((((((TS=("gestational hyperglycemia ")) OR TS=("gestational diabete* ")) OR TS=("gestational glucose ")) OR TS=("pregnancy hyperglycemia")) OR TS=("pregnancy diabete* ")) OR TS=("pregnancy glucose ")) OR TS=("maternal hyperglycemia")) OR TS=("maternal diabete* ")) OR TS=("maternal glucose") | 34931 |
| 15 | ((TS=("Pregnancy in Diabetics" )) OR TS=("Diabetes, Gestational")) OR TS=("GDM") | 32851 |
| 16 | (TS=("diabet*")) AND TS=("pregnan*") | 55279 |
| 17 | (TS=("abnormal oral glucose")) AND TS=("pregnan*") | 57 |
| 18 | (TS=("abnormal glucose")) AND TS=("pregnan*") | 653 |
| 19 | (TS=("glucose intoleran*")) AND TS=("pregnan*") | 3031 |
| 20 | #13 OR #14 OR #15 OR #16 OR #17 OR #18 OR #19 | 65273 |
| 21 | ((((((((((((((((((((((TS=("mortality")) OR TS=("death")) OR TS=("stillbirth")) OR TS=("congenital")) OR TS=("malformation")) OR TS=("anomaly")) OR TS=("impair* ")) OR TS=("disabilit*")) OR TS=("macrosomia")) OR TS=("lbw")) OR TS=("large for gestational age")) OR TS=("LGA")) OR TS=("SGA")) OR TS=("neonatal hypoglyc*")) OR TS=("neonatal hyperbili*")) OR TS=("jaundice")) OR TS=("respiratory distress syndrome")) OR TS=("RDS")) OR TS=("hyaline membrane disease")) OR TS=("HMD")) OR TS=("Apgar")) OR TS=("asphyxia")) OR TS=("apnoea") | 5411483 |
| 22 | ((((((((((((((TS=("Spontaneous miscarriage")) OR TS=("Pregnancy loss")) OR TS=("Antepartum hemorrhage")) OR TS=("Post-partum hemorrhage")) OR TS=("Induction of labour")) OR TS=("Instrumental birth")) OR TS=("Caesarean section")) OR TS=("Shoulder dystocia")) OR TS=("Eclampsia")) OR TS=("Placenta previa ")) OR TS=("Placental abruption")) OR TS=("Premature rupture of membrane")) OR TS=("Infection")) OR TS=("Amniotic embolism")) OR TS=("Hyperemesis gravidarum") | 3577841 |
| 23 | ((((((TS=("fetal-outcom*")) OR TS=("fetus outcome*")) OR TS=("obstetric* outcome*")) OR TS=("maternal-outcom*")) OR TS=("neonatal-outcom*")) OR TS=("perinatal-outcom*")) OR TS=("Maternal Outcomes") | 44900 |
| 24 | ((TS=("*Gastrointestinal Hemorrhage ")) OR TS=("Variceal bleed")) OR TS=("Pregnancy Complications") | 167957 |
| 25 | ((TS=("*Postoperative Hemorrhage ")) OR TS=("Hemorrhage ")) OR TS=("Hematemesis ") | 352408 |
| 26 | (((TS=("Abruptio Placentae ")) OR TS=("Abruptio")) OR TS=("Hypertension ")) OR TS=("*Hypertension, Pregnancy-Induced") | 731024 |
| 27 | (((TS=("Preeclampsia ")) OR TS=("Pre-Eclampsia")) OR TS=("Thrombocytopenia, Neonatal Alloimmune ")) OR TS=("Thrombocytopenia ") | 150856 |
| 28 | ((TS=("Anemia ")) OR TS=("Blood transfusion ")) OR TS=("Blood Transfusion") | 306057 |
| 29 | ((TS=("Platelet transfusion ")) OR TS=("*Platelet Transfusion")) OR TS=("Decompensation") | 26940 |
| 30 | (((TS=("*Jaundice, Neonatal ")) OR TS=("Ascites ")) OR TS=("Sepsis ")) OR TS=("sepsis") | 251353 |
| 31 | (((TS=("Maternal death ")) OR TS=("Maternal Death")) OR TS=("Maternal mortality ")) OR TS=("Maternal Mortality") | 20050 |
| 32 | (((TS=("Fetal complications")) OR TS=("*Abortion, Habitual ")) OR TS=("*Abortion, Spontaneous ")) OR TS=("*Abortion, Induced ") | 47996 |
| 33 | (TS=("Low birth weight ")) OR TS=("Infant, Low Birth Weight") | 61187 |
| 34 | (TS=("Mode of Delivery ")) OR TS=("Delivery, Obstetric") | 29577 |
| 35 | (((TS=("Cesarean section")) OR TS=("Premature Birth ")) OR TS=("Preterm delivery ")) OR TS=("*Obstetric Labor, Premature") | 110019 |
| 36 | ((TS=("Intrauterine growth restriction ")) OR TS=("Fetal Growth Retardation")) OR TS=("small for gestational age") | 41408 |
| 37 | ((TS=("*Intensive Care Units, Neonatal ")) OR TS=("NICU admission")) OR TS=("neonatal intensive care unit") | 29591 |
| 38 | (TS=("Neonatal death ")) OR TS=("Perinatal Death") | 10631 |
| 39 | #21 OR #22 OR #23 OR #24 OR #25 OR #26 OR #27 OR #28 OR #29 OR #30 OR #31 OR #32 OR #33 OR #34 OR #35 OR #36 OR #37 OR #38 | 9531819 |
| 40 | ((TS=(randomized controlled trial)) OR TS=(randomized)) OR TS=(placebo) | 1374155 |
| 41 | #11 AND #20 AND #39 AND #40 | 119 |

| **#** | **Appendix(A.4) Searches (embase)** | **Outcome** |
| --- | --- | --- |
| 1 | ('self-monitoring blood glucose'/exp OR 'self-monitoring blood glucose' OR 'blood glucose self monitoring':ab,kw OR 'glucose, blood, self-monitoring':ab,kw OR 'self-monitoring, blood glucose':ab,kw OR 'blood glucose self-monitorings':ab,kw OR 'glucose self-monitoring, blood':ab,kw OR 'glucose self-monitorings, blood':ab,kw OR 'self monitoring, blood glucose':ab,kw OR 'self-monitorings, blood glucose':ab,kw OR 'monitoring, home blood glucose':ab,kw OR 'blood sugar self-monitoring':ab,kw OR 'blood sugar self monitoring':ab,kw OR 'blood sugar self-monitorings':ab,kw OR 'self-monitoring, blood sugar':ab,kw OR 'self-monitorings, blood sugar':ab,kw OR 'sugar self-monitoring, blood':ab,kw OR 'sugar self-monitorings, blood':ab,kw OR 'home blood glucose monitoring':ab,kw OR 'glucose, blood, self monitoring':ab,kw OR 'smbg':ab,kw OR 'Continuous Glucose Monitoring'/exp OR 'Glucose Monitoring, Continuous':ab,kw OR 'Monitoring, Continuous Glucose':ab,kw OR 'Monitorings, Continuous Glucose':ab,kw OR 'Continuous Glucose Monitoring Device':ab,kw OR 'CGM Device':ab,kw OR 'CGM Devices':ab,kw OR 'Device, CGM':ab,kw OR 'Devices, CGM':ab,kw OR 'CGM':ab,kw OR 'CGMS':ab,kw) | 21968 |
| 2 | ('gestational diabetes'/exp OR 'gestational diabetes' OR 'diabetes, pregnancy-induced':ab,kw OR 'diabetes, pregnancy induced':ab,kw OR 'pregnancy-induced diabetes':ab,kw OR 'gestational diabetes':ab,kw OR 'diabetes mellitus, gestational':ab,kw OR 'gestational diabetes mellitus':ab,kw OR 'gestational hyperglycemia':ab,kw OR 'gestational diabete*':ab,kw OR 'gestational glucose':ab,kw OR 'pregnancy hyperglycemia':ab,kw OR 'pregnancy diabete*':ab,kw OR 'pregnancy glucose':ab,kw OR 'maternal hyperglycemia':ab,kw OR 'maternal diabete*':ab,kw OR 'maternal glucose':ab,kw OR 'pregnancy in diabetics':ab,kw OR 'diabetes, gestational':ab,kw OR 'gdm':ab,kw OR 'diabet* and pregnan*':ab,kw OR 'abnormal oral glucose and pregnan*':ab,kw OR 'abnormal glucose and pregnan*':ab,kw OR 'glucose intoleran* and pregnan*':ab,kw) | 63471 |
| 3 | ('perinatal outcome'/exp OR 'gastrointestinal hemorrhage':ab,kw OR 'variceal bleed':ab,kw OR 'pregnancy complications':ab,kw OR 'postoperative hemorrhage':ab,kw OR 'hemorrhage':ab,kw OR 'hematemesis':ab,kw OR 'abruptio placentae':ab,kw OR 'abruptio':ab,kw OR 'hypertension':ab,kw OR 'hypertension, pregnancy-induced':ab,kw OR 'preeclampsia':ab,kw OR 'pre-eclampsia':ab,kw OR 'thrombocytopenia, neonatal alloimmune':ab,kw OR 'thrombocytopenia':ab,kw OR 'anemia':ab,kw OR 'blood transfusion':ab,kw OR 'platelet transfusion':ab,kw OR 'decompensation':ab,kw OR 'jaundice, neonatal':ab,kw OR 'ascites':ab,kw OR 'sepsis':ab,kw OR 'maternal death':ab,kw OR 'maternal mortality':ab,kw OR 'fetal complications':ab,kw OR 'abortion, habitual':ab,kw OR 'abortion, spontaneous':ab,kw OR 'abortion, induced':ab,kw OR 'low birth weight':ab,kw OR 'infant, low birth weight':ab,kw OR 'mode of delivery':ab,kw OR 'delivery, obstetric':ab,kw OR 'cesarean section':ab,kw OR 'premature birth':ab,kw OR 'preterm delivery':ab,kw OR 'obstetric labor, premature':ab,kw OR 'intrauterine growth restriction':ab,kw OR 'fetal growth retardation':ab,kw OR 'small for gestational age':ab,kw OR 'nicu admission':ab,kw OR 'intensive care units, neonatal':ab,kw OR 'neonatal intensive care unit':ab,kw OR 'neonatal death':ab,kw OR 'perinatal death':ab,kw OR 'fetal-outcom*':ab,kw OR 'fetus outcome*':ab,kw OR 'obstetric* outcome*':ab,kw OR 'maternal-outcom*':ab,kw OR 'neonatal-outcom*':ab,kw OR 'perinatal-outcom*':ab,kw OR 'maternal outcomes':ab,kw OR 'spontaneous miscarriage':ab,kw OR 'pregnancy loss':ab,kw OR 'antepartum hemorrhage':ab,kw OR 'post-partum hemorrhage':ab,kw OR 'induction of labour':ab,kw OR 'instrumental birth':ab,kw OR 'caesarean section':ab,kw OR 'shoulder dystocia':ab,kw OR 'eclampsia':ab,kw OR 'placenta previa':ab,kw OR 'placental abruption':ab,kw OR 'premature rupture of membrane':ab,kw OR 'infection':ab,kw OR 'amniotic embolism':ab,kw OR 'hyperemesis gravidarum':ab,kw OR 'mortality':ab,kw OR 'death':ab,kw OR 'stillbirth':ab,kw OR 'congenital':ab,kw OR 'malformation':ab,kw OR 'anomaly':ab,kw OR 'impair*':ab,kw OR 'disabilit*':ab,kw OR 'macrosomia':ab,kw OR 'lbw':ab,kw OR 'large for gestational age':ab,kw OR 'lga':ab,kw OR 'sga':ab,kw OR 'neonatal hypoglyc*':ab,kw OR 'neonatal hyperbili*':ab,kw OR 'jaundice':ab,kw OR 'respiratory distress syndrome':ab,kw OR 'rds':ab,kw OR 'hyaline membrane disease':ab,kw OR 'hmd':ab,kw OR 'apgar':ab,kw OR 'asphyxia':ab,kw OR 'apnoea':ab,kw) | 6877445 |
| 4 | (randomized controlled trial):ab,ti,kw OR (randomized):ab,ti,kw OR (placebo):ab,kw | 1204659 |
| 5 | #1 AND #2 AND #3 AND #4 | 59 |

| **#** | **Searches(A.4) cochrane library** | **Outcome** |
| --- | --- | --- |
| 1 | MeSH descriptor: [Blood Glucose Self-Monitoring] explode all trees | 1580 |
| 2 | MeSH descriptor: [Diabetes, Gestational] explode all trees | 1734 |
| 3 | ("blood glucose self monitoring"):ab,ti,kw OR ("glucose, blood, self-monitoring"):ab,ti,kw OR ("self-monitoring, blood glucose"):ab,ti,kw OR ("blood glucose self-monitorings"):ab,ti,kw OR ("glucose self-monitoring, blood"):ab,ti,kw OR ("glucose self-monitorings, blood"):ab,ti,kw OR ("self monitoring, blood glucose"):ab,ti,kw OR ("self-monitorings, blood glucose"):ab,ti,kw OR ("monitoring, home blood glucose"):ab,ti,kw OR ("blood sugar self-monitoring"):ab,ti,kw OR ("blood sugar self monitoring"):ab,ti,kw OR ("blood sugar self-monitorings"):ab,ti,kw OR ("self-monitoring, blood sugar"):ab,ti,kw OR ("self-monitorings, blood sugar"):ab,ti,kw OR ("sugar self-monitoring, blood"):ab,ti,kw OR ("sugar self-monitorings, blood"):ab,ti,kw OR ("home blood glucose monitoring"):ab,ti,kw OR ("glucose, blood, self monitoring"):ab,ti,kw OR ("smbg"):ab,ti,kw OR ("Continuous Glucose Monitoring"):ab,ti,kw OR ("Glucose Monitoring, Continuous"):ab,ti,kw OR ("Monitoring, Continuous Glucose"):ab,ti,kw OR ("Monitorings, Continuous Glucose"):ab,ti,kw OR ("Continuous Glucose Monitoring Device"):ab,ti,kw OR ("CGM Device"):ab,ti,kw OR ("CGM Devices"):ab,ti,kw OR ("Device, CGM"):ab,ti,kw OR ("Devices, CGM"):ab,ti,kw OR ("CGM"):ab,ti,kw OR ("CGMS"):ab,ti,kw | 5709 |
| 4 | #1 OR #3 | 5709 |
| 15 | ("Diabetes, Pregnancy-Induced"):ab,ti,kw OR ("Diabetes, Pregnancy Induced"):ab,ti,kw OR ("Pregnancy-Induced Diabetes"):ab,ti,kw OR ("Gestational Diabetes"):ab,ti,kw OR ("Diabetes Mellitus, Gestational"):ab,ti,kw OR ("Gestational Diabetes Mellitus"):ab,ti,kw | 3831 |
| 16 | ("gestational hyperglycemia"):ab,ti,kw OR (gestational NEXT diabete* ):ab,ti,kw OR ("gestational glucose" ):ab,ti,kw OR ("pregnancy hyperglycemia"):ab,ti,kw OR (pregnancy NEXT diabete* ):ab,ti,kw OR ("pregnancy glucose" ):ab,ti,kw OR ("maternal hyperglycemia"):ab,ti,kw OR (maternal NEXT diabete* ):ab,ti,kw OR ("maternal glucose"):ab,ti,kw | 4148 |
| 17 | ("Pregnancy in Diabetics" ):ab,ti,kw OR ("Diabetes, Gestational"):ab,ti,kw OR (GDM):ab,ti,kw | 2906 |
| 18 | (diabet*):ab,ti,kw AND (pregnan*):ab,ti,kw | 7839 |
| 19 | ("abnormal oral glucose"):ab,ti,kw AND ( pregnan*):ab,ti,kw | 9 |
| 20 | ("abnormal glucose"):ab,ti,kw AND ( pregnan*):ab,ti,kw | 74 |
| 21 | (glucose NEXT intoleran*):ab,ti,kw AND ( pregnan*):ab,ti,kw | 236 |
| 22 | #2 OR #15 OR #16 OR #17 OR #18 OR #19 OR #20 OR #21 | 8575 |
| 23 | ("Pregnancy Complications"):ab,ti,kw OR (*Postoperative Hemorrhage):ab,ti,kw OR ("Hemorrhage"):ab,ti,kw | 43680 |
| 24 | ("Spontaneous miscarriage"):ab,ti,kw OR ("Pregnancy loss"):ab,ti,kw OR ("Antepartum hemorrhage"):ab,ti,kw OR ("Post-partum hemorrhage"):ab,ti,kw OR ("Induction of labour"):ab,ti,kw OR ("Instrumental birth"):ab,ti,kw OR ("Caesarean section"):ab,ti,kw OR ("Shoulder dystocia"):ab,ti,kw OR ("Eclampsia"):ab,ti,kw OR ("Placenta previa"):ab,ti,kw OR ("Placental abruption"):ab,ti,kw OR ("Premature rupture of membrane"):ab,ti,kw OR ("Infection"):ab,ti,kw OR ("Amniotic embolism"):ab,ti,kw OR ("Hyperemesis gravidarum"):ab,ti,kw | 132768 |
| 25 | (*Gastrointestinal Hemorrhage):ab,ti,kw OR ("Variceal bleed"):ab,ti,kw | 5671 |
| 26 | (fetal-outcom*):ab,ti,kw OR (fetus NEXT outcome*):ab,ti,kw OR (obstetric* NEXT outcome*):ab,ti,kw OR (maternal NEXT-outcom*):ab,ti,kw OR (neonatal-outcom*):ab,ti,kw OR (perinatal-outcom*):ab,ti,kw OR (Maternal NEXT Outcomes):ab,ti,kw | 6919 |
| 27 | ("mortality"):ab,ti,kw OR ("death"):ab,ti,kw OR ("stillbirth"):ab,ti,kw OR ("congenital"):ab,ti,kw OR ("malformation"):ab,ti,kw OR ("anomaly"):ab,ti,kw OR (impair*):ab,ti,kw OR (disabilit*):ab,ti,kw OR ("macrosomia"):ab,ti,kw OR ("lbw"):ab,ti,kw OR ("large for gestational age"):ab,ti,kw OR ("LGA"):ab,ti,kw OR ("SGA"):ab,ti,kw OR (neonatal hypoglyc*):ab,ti,kw OR (neonatal hyperbili*):ab,ti,kw OR ("jaundice"):ab,ti,kw OR ("respiratory distress syndrome"):ab,ti,kw OR ("RDS"):ab,ti,kw OR ("hyaline membrane disease"):ab,ti,kw OR ("HMD"):ab,ti,kw OR ("Apgar"):ab,ti,kw OR ("asphyxia"):ab,ti,kw OR ("apnoea"):ab,ti,kw | 333609 |
| 28 | ("Hematemesis"):ab,ti,kw OR ("Abruptio Placentae"):ab,ti,kw OR ("Abruptio"):ab,ti,kw | 553 |
| 29 | ("Hypertension"):ab,ti,kw OR (*Hypertension, Pregnancy-Induced):ab,ti,kw OR ("Preeclampsia"):ab,ti,kw OR ("Pre-Eclampsia"):ab,ti,kw | 28617 |
| 30 | ("Thrombocytopenia, Neonatal Alloimmune"):ab,ti,kw OR ("Thrombocytopenia"):ab,ti,kw OR ("Anemia"):ab,ti,kw | 32664 |
| 31 | ("Blood transfusion"):ab,ti,kw OR ("Platelet transfusion"):ab,ti,kw OR ("Blood Transfusion"):ab,ti,kw OR (*Platelet Transfusion):ab,ti,kw | 12478 |
| 32 | ("Decompensation"):ab,ti,kw OR (*Jaundice, Neonatal):ab,ti,kw | 1490 |
| 33 | ("Ascites"):ab,ti,kw OR ("Sepsis"):ab,ti,kw OR ("sepsis"):ab,ti,kw | 17471 |
| 34 | ("Maternal death"):ab,ti,kw OR ("Maternal Death"):ab,ti,kw OR ("Maternal mortality"):ab,ti,kw OR ("Maternal Mortality"):ab,ti,kw OR ("Fetal complications"):ab,ti,kw | 1655 |
| 35 | (*Abortion, Habitual):ab,ti,kw OR (*Abortion, Spontaneous):ab,ti,kw OR (*Abortion, Induced):ab,ti,kw | 0 |
| 36 | ("Low birth weight"):ab,ti,kw OR ("Infant, Low Birth Weight"):ab,ti,kw | 6076 |
| 37 | ("Mode of Delivery"):ab,ti,kw OR ("Delivery, Obstetric"):ab,ti,kw | 3764 |
| 38 | ("Cesarean section"):ab,ti,kw OR ("Premature Birth"):ab,ti,kw OR ("Preterm delivery"):ab,ti,kw OR (*Obstetric Labor, Premature):ab,ti,kw | 20422 |
| 39 | ("Intrauterine growth restriction"):ab,ti,kw OR ("Fetal Growth Retardation"):ab,ti,kw | 1014 |
| 40 | ("small for gestational age"):ab,ti,kw | 1355 |
| 41 | ("NICU admission"):ab,ti,kw OR (*Intensive Care Units, Neonatal):ab,ti,kw OR ("neonatal intensive care unit"):ab,ti,kw | 5434 |
| 42 | ("Neonatal death"):ab,ti,kw OR ("Perinatal Death"):ab,ti,kw | 1428 |
| 43 | #23 OR #24 OR #25 OR #26 OR #27 OR #28 OR #29 OR #30 OR #31 OR #32 OR #33 OR #34 OR #35 OR #36 OR #37 OR #38 OR #39 OR #40 OR #41 OR #42 | 548145 |
| 44 | (randomized controlled trial):ab,ti,kw OR (randomized):ab,ti,kw OR (placebo):ab,ti,kw | 1316197 |
| 45 | #4 AND #22 AND #43 AND #44 | 156 |
| 46 |  |  |
