# Supplementary material for: A systematic review and meta-analysis of the effects of continuous glucose monitoring compared to self-monitoring of blood glucose on glycemic control and maternal and infant outcomes in women with gestational diabetes mellitus
Source: BMC Pregnancy Childbirth. 2026 Aug 8;26:1272. doi: 10.1186/s12884-026-09769-9 (PMC13733083; doi:10.1186/s12884-026-09769-9)
Supplement: Supplementary file 2 [file 12884_2026_9769_MOESM2_ESM.docx]

eTable 1 Summary of effect sizes, confidence intervals, P-values, and heterogeneity estimates for outcomes across subgroups

| Subgroup analysis | | | References |  | Sample(CGM Group) |  | Sample(SMBG Group) |  | Effect estimates (95% CI) |  | P value |  | I^2^ |
| --- | --- | --- | --- | --- | --- | --- | --- | --- | --- | --- | --- | --- | --- |
| Intervention | |  |  |  |  |  |  |  |  |  |  |  |  |
| glycosylated hemoglobin | r-CGM | | Qiong  M.Y. |  | 113 |  | 117 |  | -0.18(-0.44, 0.08) |  | 0.180 |  | 0.0% |
|  | rt-CGM | | Alfadhli |  | 60 |  | 62 |  | -0.70(-1.07, -0.34) |  | 0.000 |  | 0.0% |
| the number of people treated with hypoglycemic drugs | r-CGM | | Kirsimarja K.  Qiong  M.Y. |  | 149 |  | 154 |  | 2.13(1.27,3.58) |  | 0.004 |  | 19.5% |
|  | rt-CGM | | Alfadhli |  | 60 |  | 62 |  | 1.09(0.64, 1.87) |  | 0.752 |  | 0.0% |
| hyperbilirubinemia | r-CGM | | Kirsimarja K.  M.Y. |  | 98 |  | 99 |  | 0.79(0.38, 1.66) |  | 0.537 |  | 0.0% |
|  | rt-CGM | | Alfadhli |  | 60 |  | 62 |  | 1.55(0.59, 4.09) |  | 0.376 |  | 0.0% |
| macrosomia | r-CGM | | Kirsimarja K.  Qiong  M.Y. |  | 149 |  | 154 |  | 0.86(0.37, 2.00) |  | 0.728 |  | 0.0% |
|  | rt-CGM | | Alfadhli |  | 60 |  | 62 |  | 0.34(0.01, 8.29) |  | 0.574 |  | 0.0% |
| neonatal NICU | r-CGM | | Kirsimarja K.  M.Y. |  | 98 |  | 99 |  | 0.71(0.38, 1.32) |  | 0.277 |  | 0.0% |
|  | rt-CGM | | Alfadhli |  | 60 |  | 62 |  | 1.21(0.72, 2.03) |  | 0.481 |  | 0.0% |
| neonatal hypoglycemic | r-CGM | | Kirsimarja K.  Qiong  M.Y. |  | 149 |  | 154 |  | 0.81(0.37, 1.76) |  | 0.590 |  | 0.0% |
|  | rt-CGM | | Alfadhli |  | 60 |  | 62 |  | 1.72(0.67, 4.44) |  | 0.261 |  | 0.0% |
| birth weight | r-CGM | | Kirsimarja K.  Qiong  M.Y. |  | 149 |  | 154 |  | -0.30(-0.54, -0.06) |  | 0.009 |  | 8.6% |
|  | rt-CGM | | Alfadhli |  | 60 |  | 62 |  | -0.32(-0.67, 0.04) |  | 0.082 |  | 0.0% |
