# Supplementary material for: A systematic review and meta-analysis of the effects of continuous glucose monitoring compared to self-monitoring of blood glucose on glycemic control and maternal and infant outcomes in women with gestational diabetes mellitus
Source: BMC Pregnancy Childbirth. 2026 Aug 8;26:1272. doi: 10.1186/s12884-026-09769-9 (PMC13733083; doi:10.1186/s12884-026-09769-9)
Supplement: Supplementary file 3 [file 12884_2026_9769_MOESM3_ESM.docx]

**eFigure 1.** Comparison of baseline characteristics between continuous glucose monitoring (CGM) and self-monitoring of blood glucose (SMBG) groups in included trials.

*Displayed characteristics include maternal age, pregnancy body mass index (BMI), oral glucose tolerance test (OGTT) at 0, 1, and 2 hours, haemoglobin A1c (HbA1c), high-density lipoprotein (HDL), low-density lipoprotein (LDL), and gestational age at delivery. SMD: standardized mean difference; 95% CI: 95% confidence interval.*

**eFigure 2.** Number of people treated with hypoglycemic drugs in CGM versus SMBG groups.

*CGM: Continuous Glucose Monitoring; SMBG：Self-monitoring of blood glucose; 95% CI: 95% Confidence Interval; Risk Ratio: RR.*

**eFigure 3.** HbA1c and glucose-lowering drug doses in CGM versus SMBG groups.

Haemoglobin: haemoglobin A1c; *CGM: Continuous Glucose Monitoring; SMBG: Self-monitoring of blood glucose; 95% CI: 95% Confidence Interval; SMD: Standardized Mean Difference;*

**eFigure 4.** Caesarean section rates in CGM versus SMBG groups.

*CGM: Continuous Glucose Monitoring; SMBG: Self-monitoring of blood glucose; 95% CI: 95% Confidence Interval; Risk Ratio: RR.*

**eFigure 5.** Neonatal outcomes(binary) in CGM versus SMBG groups.

*CGM: Continuous Glucose Monitoring; SMBG：Self-monitoring of blood glucose; 95% CI: 95% Confidence Interval; Risk Ratio: RR. Neonatal outcomes assessed included macrosomia, LGA, SGA, hyperbilirubinemia, neonatal hypoglycemia, neonatal intensive care unit (NICU) admission.*

**eFigure 6.** Neonatal outcomes(continuous) in CGM versus SMBG groups.

*CGM: Continuous Glucose Monitoring; SMBG: Self-monitoring of blood glucose; 95% CI: 95% Confidence Interval; SMD: Standardized Mean Difference;*

**eFigure 7-A.** Neonatal Hyperbilirubinemia in Retrospective and Real-Time CGM Subgroups Versus SMBG Group

**eFigure 7-B.** Macrosomia in Retrospective and Real-Time CGM Subgroups Versus SMBG Group

**eFigure 7-C.** Neonatal Hypoglycemia in Retrospective and Real-Time CGM Subgroups Versus SMBG Group

**eFigure 7-D.** NICU Admission in Retrospective and Real-Time CGM Subgroups Versus SMBG Group

retro: retrospective continuous glucose monitoring; realm: real-time continuous glucose monitoring; SMBG：Self-monitoring of blood glucose; 95% CI: 95% Confidence Interval ; Risk Ratio: RR.

**eFigure 8**. Birth Weight in the Retrospective CGM and Real-Time CGM Subgroups Versus the Self-Monitoring of Blood Glucose Group

*retro: retrospective continuous glucose monitoring; realm: real-time continuous glucose monitoring; SMBG：Self-monitoring of blood glucose; 95% CI: 95% Confidence Interval ; SMD: Standardized Mean Difference.*

**eFigure 9-A. Sensitivity analysis of GWG-Excessive results**

| **Study omitted** | **Estimate** | **[95% Conf.** | **Interval]** |
| --- | --- | --- | --- |
|  |  |  |  |
| Qiong | 0.55172414 | 0.33509457 | 0.90839887 |
| M.Y. | 0.59139782 | 0.37616596 | 0.92977959 |
|  |  |  |  |
| Combined | 0.57314118 | 0.4099589 | 0.80127743 |

**eFigure 9-B. Sensitivity analysis of GWG-Appropriate results**

| **Study omitted** | **Estimate** | **[95% Conf.** | **Interval]** |
| --- | --- | --- | --- |
|  |  |  |  |
| Qiong | 1.48 | 1.026935 | 2.1329491 |
| M.Y. | 1.643324 | 1.1045903 | 2.4448102 |
|  |  |  |  |
| Combined | 1.5527492 | 1.1865738 | 2.0319258 |

**eFigure 9-C. Sensitivity analysis of GWG-Inadequate results**

| **Study omitted** | **Estimate** | **[95% Conf.** | **Interval]** |
| --- | --- | --- | --- |
|  |  |  |  |
| Qiong | 1.125 | 0.46433806 | 2.7256544 |
| M.Y. | 0.71895427 | 0.12516615 | 4.1296725 |
|  |  |  |  |
| Combined | 1.0268077 | 0.46622946 | 2.2614058 |

**eFigure 9-D. Sensitivity analysis of outcomes in the number of people treated with glucose-lowering drugs**

| **Study omitted** | **Estimate** | **[95% Conf.** | **Interval]** |
| --- | --- | --- | --- |
|  |  |  |  |
| Kirsimarja K. | 1.4142449 | 0.87024093 | 2.298316 |
| Qiong | 1.4607338 | 0.78378153 | 2.7223701 |
| M.Y. | 1.908823 | 0.90998298 | 4.0040369 |
| Alfadhli | 2.1001823 | 1.1612622 | 3.7982519 |
|  |  |  |  |
| Combined | 1.6635636 | 0.97586798 | 2.8358793 |

**eFigure 9-E. Sensitivity analysis of HbA1c results**

| **Study omitted** | **Estimate** | **[95% Conf.** | **Interval]** |
| --- | --- | --- | --- |
|  |  |  |  |
| Qiong | -0.39954808 | -0.99334031 | 0.19424418 |
| M.Y. | -0.49130905 | -0.91674984 | -0.06586823 |
| Alfadhli | -0.17744412 | -0.43663877 | 0.08175053 |
|  |  |  |  |
| Combined | -0.35657163 | -0.71496821 | 0.00182495 |
